# Comparative Outcomes and Predictive Assessment of Trifecta in Open, Laparoscopic, and Robotic-Assisted Partial Nephrectomy Cases with Renal Cell Carcinoma: A 10-Year Experience at Ramathibodi Hospital

**DOI:** 10.2147/RRU.S316824

**Published:** 2021-06-28

**Authors:** Chaichant Soisrithong, Pokket Sirisreetreerux, Premsant Sangkum, Kittinut Kijvikai, Wit Viseshsindh, Wisoot Kongchareonsombat, Charoen Leenanupunth, Wachira Kochakarn, Pocharapong Jenjitranant

**Affiliations:** 1 Division of Urology, Department of Surgery, Faculty of Medicine Ramathibodi Hospital, Mahidol University, Bangkok, Thailand

**Keywords:** partial nephrectomy, trifecta, Thai, laparoscopy, robotic-assisted partial nephrectomy

## Abstract

**Purpose:**

To compare perioperative and trifecta outcomes of open partial nephrectomy (OPN), laparoscopic partial nephrectomy (LPN), and robotic-assisted laparoscopic partial nephrectomy (RPN) in patients with small renal mass at Ramathibodi Hospital, and to determine predictive factors in connection with trifecta.

**Methods:**

We retrospectively reviewed 141 patients who underwent partial nephrectomy by eight experienced surgeons from January 2009 to December 2018. Baseline preoperative characteristics, postoperative and trifecta outcomes of the three treatment modalities were compared and analyzed. Univariate analysis was performed to determine predictive factors for trifecta achievement.

**Results:**

A total of 70 patients had complete data available. Eighteen OPN, 11 LPN and 41 RPN cases were identified and reviewed. All preoperative and perioperative parameters were similar, except for operative time, which was significantly shorter in the OPN group compared with those undergoing LPN and RPN (135 vs 189 and 225 min, respectively; p-value = 0.001). Of these 70 patients, 59 were deemed eligible for and included in trifecta analysis, which revealed similar trifecta outcomes (64.29%, 45.45%, and 64.71% in the OPN, LPN, and RPN groups, respectively; p-value = 0.388). Univariate analysis showed that length of hospital stay was a negative associated factor for trifecta achievement (p-value = 0.007, 95% CI = 0.619 (0.44–0.88)).

**Conclusion:**

Although OPN displayed the shortest operative time, the trifecta achievement rate was not significantly different among the three groups. The sole parameter, which was negatively associated with trifecta outcome achievement, was the length of hospital stay.

## Introduction

Renal cell carcinoma (RCC) accounts for 2% to 3% of all adult neoplasms and is responsible for approximately 64,000 newly diagnosed cases and 14,400 deaths each year in the United States.^1^ In contrast, the incidence and mortality rates are approximately 50% lower in Asian-Americans/Pacific Islanders.^1^ Renal masses may be either malignant or benign with those smaller than 4 cm being considered benign in 20% to 30% of the cases.^2^,^3^ Many clinical T1a cancers have indolent biology whose likelihood of malignancy increases with each 1-cm increase in diameter.^4^,^5^ Common symptoms are flank pain, a palpable mass, and hematuria.

Advances in imaging techniques have improved the incidental detection of renal masses, particularly small renal masses.^2^,^6^ Partial nephrectomy (PN) has increasingly become a standard therapeutic option for patients with renal masses, showing equivalent oncologic outcomes compared with radical nephrectomy.^7^ The major advantages of PN over RN include improvement of morbidity and mortality, reduced urologic complications, and decreased risk of development or progression of chronic kidney disease (CKD).^8–12^ According to the 2017 American Urological Association guideline, partial nephrectomy is recommended for patients with cT1a renal masses and other conditions in which radical nephrectomy may compromise future renal function.^9^

Historically, open partial nephrectomy (OPN) was performed in all cases. However, laparoscopic partial nephrectomy (LPN) and robotic-assisted laparoscopic partial nephrectomy (RPN) are now being increasingly implemented worldwide.^13^ The first robot-assisted laparoscopic prostatectomy was performed in 2000,^14^ and robotic-assisted renal surgery was performed thereafter. RPN has been performed in Ramathibodi Hospital since 2015. Despite the widespread adoption of RPN, the available body of literature provides debatable evidence as to which surgical technique offers the most successful and superior outcomes.

The concept of the “trifecta” outcome has recently been used to evaluate surgical proficiency and includes negative surgical margins, no severe surgical complications, and 90% preservation of the estimated glomerular filtration rate (eGFR) postoperatively.^15^ However, studies that have directly compared trifecta achievement of OPN, LPN and RPN remain limited. Also, the factors that affect the trifecta outcome are unclear. This study was therefore conducted to compare preoperative, postoperative, and trifecta outcomes of OPN, LPN, and RPN, and to identify predictive factors for trifecta outcome achievement. Comparison of these three surgical approaches may ultimately contribute to a better consensus on the clinical variables associated with favorable outcomes after partial nephrectomy.

## Materials and Methods

### Study Population

We retrospectively reviewed the medical records of all patients who underwent OPN, LPN, and RPN at Ramathibodi Hospital from January 2009 to December 2018. All of the patients with the tumor size of 6 cm or less who underwent partial nephrectomy in our institution and the pathological confirm of renal cell carcinoma were included in the study. Patients with incomplete data and pathologically benign lesions were excluded. Regarding the patients with tumor size more than 6 cm in diameter, radical nephrectomy is the modality of choice as per our institutional guideline. Informed consents were obtained from all subjects or, if subjects were under 18, from a parent and/or legal guardian after obtaining the ethical approval from the Committee on Human Rights Related to Research involving Human Subjects, Faculty of Medicine, Ramathibodi Hospital (MURA2019/1183).

### Surgical Techniques

The selection of the surgical approach was determined by the complexity of the tumors, surgeon preference, and financial affordability of the patients. OPN were performed by 8 consultant urologists. The operative steps of OPN included resection of perirenal fat, maintaining fat above the tumor, en bloc hilar clamping with or without renal cooling by crushed ice, cold excision of the tumor, suturing of the collecting system and renorrhaphy. LPN and RPN were performed by 4 consultant urologists. Both the LPN and RPN procedures featured similar essential steps which were resection of perirenal fat, maintaining fat above the tumor, using of laparoscopic ultrasound to determine the edge of the tumor, en bloc hilar clamping without the cooling method, suturing of the collecting system and renorrhaphy.

### Data Collection

The patients’ baseline characteristics along with disease- and treatment-related parameters were collected, including age, sex, body weight, height, body mass index, previous abdominal surgery, the American Society of Anesthesiologists classification, underlying disease, size of renal mass, RENAL nephrometry score (radius, endophytic vs exophytic, nearness to collecting system, anterior or posterior, and location relative to polar line), surgical approach (OPN, LPN, or RPN), tumor pathology, and preoperative serum creatinine concentration.

Perioperative and postoperative data were also collected, including the operative time (skin incision to skin closure time), estimated blood loss (EBL), blood transfusion, warm ischemia time, cold ischemia time, length of hospital stay (LOS), 1-year postoperative creatinine concentration, estimated glomerular filtration rate (eGFR), and perioperative and postoperative complications. Trifecta outcome achievement was defined as the combination of negative surgical margins, no severe perioperative complications (Clavien–Dindo grade 0–2), and a postoperative eGFR of ≥90% of the preoperative eGFR. All methods were carried out in accordance with relevant guidelines and regulations.

### Statistical Analysis

The patients’ characteristics, preoperative, perioperative, postoperative, and trifecta outcomes were compared among the three operative techniques using one-way analysis of variance or the Kruskal–Wallis test for continuous variables and the chi-square test for categorical variables. Baseline patients characteristic and perioperative parameter were compared between patients who did and did not achieve the trifecta outcome using Student’s *t*-test or the Mann–Whitney test for continuous variables and the chi-square test or Fisher’s exact test for categorical variables. Predictive factors associated with trifecta outcome achievement were analysed using logistic regression model. All statistical analyses were performed with Stata v.14 (StataCorp, College Station, TX, USA). Statistical significance was defined as a p-value of <0.05.

## Results

Between 2009 and 2018, there were 70 patients with small renal mass with pathological confirm of RCC included in our study. Among 70 patients, 18 underwent OPN (25.71%), 11 LPN (15.71%) and 41 RPN (58.57%). The patients’ demographics and preoperative characteristics are summarized in Table 1. Baseline patient characteristics including gender, age, body mass index, underlying disease, previous abdominal surgery, the American Society of Anesthesiologists classification, tumor size on imaging and preoperative eGFR levels were similar. Likewise, the tumor complexity according to the RENAL nephrometry score was not significantly different among the three groups.

**Table 1:** Patient Characteristics According to the Type of Operation

Patient Characteristics	Total	OPN	LPN	RAPN	p-value
	(n=70)	(n=18)	(n=11)	(n=41)	
Gender, n(%)					
Male	50 (71.43)	13 (72.22)	7 (63.64)	30 (73.17)	0.821
Female	20 (28.57)	5 (27.78)	4 (36.36)	11 (26.83)	
Age (years), mean±SD	58.03+13.27	55.50+15.26	60.91+8.44	58.36+13.48	0.155
BMI (kg/m^2^), mean±SD	25.82+4.63	24.77+4.77	27.74+5.20	25.77+4.37	0.752
Underlying disease, n(%)					
DM	13 (18.57)	3 (16.67)	3 (27.27)	7 (17.07)	0.721
HT	45 (64.29)	13 (72.22)	8 (72.73)	24 (58.54)	0.490
DLP	33 (47.14)	6 (33.33)	6 (54.55)	21 (51.22)	0.388
CKD	41 (58.57)	11 (61.11)	7 (63.64)	23 (56.10)	0.875
Single kidney, n(%)	7 (10.00)	2 (11.11)	0	5 (12.20)	0.734
Previous abdominal surgery, n(%)					
No	43 (61.43)	11 (61.11)	7 (63.64)	25 (60.98)	0.987
Yes	27 (38.57)	7 (38.89)	4 (36.36)	16 (39.02)	
ASA class, n(%)					
Class I	9 (12.86)	0	2 (18.18)	7 (17.07)	0.607
Class II	23 (32.86)	7 (38.89)	3 (27.27)	13 (31.71)	
Class III	37 (52.86)	11 (61.11)	6 (54.55)	20 (48.78)	
Class IV	1 (1.43)	0	0	1 (2.44)	
Side, n(%)					
Left	40 (57.14)	10 (55.56)	5 (45.45)	25 (60.98)	0.645
Right	30 (42.86)	8 (44.44)	6 (54.55)	16 (39.02)	
RENAL score, n(%)					
Min–6	26 (37.14)	4 (22.22)	6 (54.55)	16 (39.02)	0.153
7–9	40 (57.14)	14 (77.78)	5 (45.45)	21 (51.22)	
10–12	4 (5.71)	0	0	4 (9.76)	
Renal cell carcinoma, n(%) n=68					
Clear cell	55 (80.88)	12 (75.00)	8 (72.73)	35 (85.37)	0.279
Papillary	6 (8.82)	1 (6.25)	1 (9.09)	4 (9.76)	
Chromophobe	4 (5.88)	2 (12.50)	2 (18.18)	0	
Other	3 (4.41)	1 (6.25)	0	2 (4.88)	
Preoperative eGFR, mean±SD	77.14+25.59	75.40+30.90	79.67+17.84	77.22+25.32	0.188

The perioperative and postoperative outcomes are shown in Table 2. The OPN, LPN and RPN groups showed similar LOS, rate of conversion to radical nephrectomy, estimated blood loss, ischemic time and margin rates. The operative time was significantly shorter in the OPN group compared to the LPN and RPN groups (135 vs 189 and 225 min, respectively; p-value = 0.001). The proportion of patients who required PRC transfusion during the operation was also significantly higher in the OPN group (33%; p-value =0.033) than in the LPN and RPN groups (9.09% and 7.32%, respectively). Post-operatively, there was no significant difference in the rate of complications (defined as per Clavien grading) as well as in the 1-year median eGFR.

**Table 2:** Perioperative and Postoperative Outcomes

Outcomes	Total	OPN	LPN	RAPN	p-value
	(n=70)	(n=18)	(n=11)	(n=41)	
Length of hospital stay (days), median (IQR)	6 (5, 7)	5 (5, 7)	6 (5, 8)	6 (5, 7)	0.621
Conversion to RN, n(%)					
No	66 (94.29)	18 (100)	11 (100)	37 (90.24)	0.349
Yes	4 (5.71)	0	0	4 (9.76)	
Estimated blood loss(mL), median(IQR)	300 (200, 550)	400 (200, 700)	250 (50, 600)	300 (200, 450)	0.154
Ischemic time (min), median(IQR) n=66	26 (20, 35)	28 (22, 46)	31 (23, 35)	25 (20, 30)	0.326
Operative time(min), mean±SD n=69	196±69	135±84	189±61	225±41	0.001
Perioperative PRC, n(%)					
Yes	10 (14.29)	6 (33.33)	1 (9.09)	3 (7.32)	0.033
No	60 (85.71)	12 (66.67)	10 (90.91)	38 (92.68)	
Margin, n(%) n=68					
Positive	2 (2.94)	0	0	2 (4.88)	0.999
Negative	66 (97.06)	16 (100)	11 (100)	39 (95.12)	
Postoperative complications (Clavien–Dindo), n(%)					
Grade 0–1	52 (74.29)	15 (83.33)	10 (90.91)	27 (65.85)	0.417
Grade 2	13 (18.57)	1 (5.56)	1 (9.09)	11 (26.83)	
Grade 3a	2 (2.86)	1 (5.56)	0	1 (2.44)	
Grade 4a	1 (1.43)	0	0	1 (2.44)	
Grade 4b	2 (2.86)	1 (5.56)	0	1 (2.44)	
eGFR at 1 year, median (IQR)	91 (81, 99)	90 (84, 103)	87 (79, 102)	94 (78, 98)	0.841

The trifecta outcomes were then assessed and compared among the 3 groups (Table 3). The parameters, which included the ≤10% decrease in the postoperative estimated GFR value at one-year follow-up (43.75% for OPN, 54.55% for LPN, and 32.35% for RPN; p-value = 0.388), negative surgical margin (100% for OPN, 100% for LPN, and 95.12% for RPN; p-value = 0.999), and absence of severe complications (Clavien-Dindo grades 3–5) (88.89% for OPN, 100% for LPN, and 92.68% for RPN; p-value =0.675) were not statistically different among the three groups. According to the 59 patients who had complete trifecta outcome data, the percentage of the patients who achieved all of the three trifecta outcomes were similar across the three surgical modalities (64.29% for OPN, 45.45% for LPN, and 64.71% for RPN; p-value = 0.502)

**Table 3:** Number of Patients Who Achieved Trifecta Outcomes and Each Domain of Trifecta Achievement

Data	Total	OPN	LPN	RAPN	p-value
	(n=70)	(n=18)	(n=11)	(n=41)	
Achieved 90% eGFR at 1 year, n(%) n=61					
<90% No	37 (60.66)	9 (56.25)	5 (45.45)	23 (67.65)	0.388
≥90% Yes	24 (39.34)	7 (43.75)	6 (54.55)	11 (32.35)	
Margin, n(%) n=68					
Negative	66 (97.06)	16 (100)	11 (100)	39 (95.12)	0.999
Positive	2 (2.94)	0	0	2 (4.88)	
Severe complications (Clavien–Dindo grade 3–5), n(%)					
No	65 (92.86)	16 (88.89)	11 (100)	38 (92.68)	0.675
Yes	5 (7.14)	2 (11.11)	0	3 (7.32)	
Trifecta outcome (n=59)					
No	23 (38.98)	5 (35.71)	6 (54.55)	12 (35.29)	0.502
Yes	36 (61.02)	9 (64.29)	5 (45.45)	22 (64.71)	

Fifty-nine patients had complete data of trifecta outcome. Patient characteristics were compared between the 36 patients who could achieve all three trifecta outcomes and the 23 patients who could not in (Table 4). There was no statistically significant difference between the two groups in all factors except for length of hospital stay (5–6 days in the trifecta group vs 6–10 days in the non-trifecta group; p-value = 0.006). Interestingly, the percentage of the patients who had the underlying disease of chronic kidney disease seemed to be different between the two groups (73.91% in the non-trifecta group vs 50% in the trifecta group); however, this was not meaningfully different in terms of statistics (p-value = 0.068).

**Table 4:** Patients and Perioperative Factors Associated with Trifecta Outcome Achievement

Patient Characteristics	Total	Non-Trifecta	Trifecta	p-value	Univariate OR (95% CI)	p-value
	(n=59)	(n=23)	(n=36)			
Gender, n(%)						
Male	44 (74.58)	19 (82.61)	25 (69.44)	0.257	1	
Female	15 (25.42)	4 (17.39)	11 (30.56)		2.09 (0.58–7.59)	0.263
Age (years), mean±SD	59.27+12.02	60.09+12.12	58.75+12.09	0.681	0.99 (0.95–1.04)	0.675
BMI (kg/m^2^), mean±SD	25.93+5.06	26.55+4.27	25.54+5.06	0.433	0.96 (0.85–1.07)	0.427
Underlying disease, n(%)						
DM	10 (16.95)	5 (21.74)	5 (13.89)	0.490	0.58 (0.15–2.28)	0.436
HT	38 (64.41)	15 (65.22)	23 (63.89)	0.917	0.93 (0.32–2.82)	0.917
DLP	28 (47.46)	11 (47.83)	17 (47.22)	0.964	0.98 (0.34–2.78)	0.964
CKD	35 (59.32)	17 (73.91)	18 (50.00)	0.068	0.35 (0.11–1.10)	0.073
Single kidney, n (%)	5 (8.47)	1 (4.35)	4 (11.11)	0.639	2.750 (0.29–26.29)	0.380
Previous abdominal surgery, n (%)						
No	35 (59.32)	14 (60.87)	21 (58.33)	0.847	1	
Yes	24 (40.68)	9 (39.13)	15 (41.67)		1.11 (0.38–3.23)	0.847
ASA class, n (%)						
Class I	9 (15.25)	3 (13.04)	6 (16.67)	0.333	1	
Class II	19 (32.20)	10 (43.48)	9 (25.00)		0.45 (0.09–225)	0.344
Class III	31 (52.54)	10 (43.48)	21 (58.33)		1.05 (0.22–5.08)	0.952
Side, n (%)						
Left	31 (52.54)	11 (47.83)	20 (55.56)	0.562	1	
Right	28 (47.46)	12 (52.17)	16 (44.44)		0.73 (0.26–2.09)	0.562
Size (cm), median (IQR)	2.7 (2.2, 3.9)	2.9 (2.2, 4.3)	2.7 (2.1, 3.5)	0.240	0.70 (0.46–1.09)	0.114
RENAL score, median (IQR)	7 (6, 8)	7 (6, 9)	7 (6, 8)	0.674	0.91 (0.67–1.24)	0.558
RENAL score group, n (%)						
Min–6	22 (37.29)	8 (34.78)	14 (38.89)	0.914		
7–9	35 (59.32)	14 (60.87)	21 (58.33)			
10–12	2 (3.39)	1 (4.35)	1 (2.78)			
Length of hospital stay (days), median (IQR)	6 (5, 7)	6 (6, 10)	6 (5, 6)	0.006	0.62 (0.44–0.88)	0.007
Ischemic time (min), median (IQR) n=56	26 (20, 35)	30 (20, 35)	25 (22, 35)	0.925	0.99 (0.94–1.03)	0.470
Operative time (min), mean±SD n=58	197+65	210+80	188+53	0.254	0.99 (0.98–1.00)	0.216
Perioperative PRC, n (%)						
No	50 (84.75)	21 (91.30)	29 (80.56)	0.263		
Yes	9 (15.25)	2 (8.70)	7 (19.44)			
Renal cell carcinoma, n (%)						
Clear cell	46 (77.97)	16 (69.57)	30 (83.33)	0.623		
Papillary	6 (10.17)	3 (13.04)	3 (8.33)			
Chromophobe	4 (6.78)	2 (8.70)	2 (5.56)			
Other	3 (5.08)	2 (8.70)	1 (2.78)			
Preoperative eGFR, mean±SD	77.51±24.05	72.91±20.79	80.46±25.77	0.243	1.013 (0.99–1.04)	0.241
Operation type, n (%)						
OPN	14 (23.73)	5 (21.74)	9 (25.00)	0.502	1	
LPN	11 (18.64)	6 (26.09)	5 (13.89)		0.463 (0.09–2.32)	0.350
RAPN	34 (57.63)	12 (52.17)	22 (61.11)		1.019 (0.28–3.74)	0.978

According to the univariable analysis of associated factors for the achievement of trifecta as shown in Table 4, LOS was again the only factor that was statistically different (OR 0.619, 95% CI 0.44, 0.88, p-value= 0.007). Chronic kidney disease was not considered to be the associated factor (OR 0.353, 95% CI 0.11, 1.10, p-value =0.073), nor were other factors as listed in the table. With regard to negative perioperative outcomes, there was no injury to major vessels or other abdominal organs in any operative modalities. Nevertheless, four patients who underwent RPN required conversion to radical nephrectomy because of high mass complexity (RENAL scores of 10P, 11A, and 10P) and mass involvement of the renal hilum in one patient (RENAL score of 6P) (Table 2). Only one patient out of the four patients who had the RENAL score of 10–12 (10P) successfully underwent RPN without conversion to radical nephrectomy (Table 1). No patient in LPN group was converted to radical nephrectomy. There was no conversion from LPN or RPN to open surgery (Table 2).

As for postoperative complications after OPN, two patients required emergency intervention. The first patient had grade 3a postoperative arteriovenous fistula (AVF) and subsequently underwent successful renal angiogram with embolization. The second patient had grade 4b postoperative cardiac arrest with atrial fibrillation requiring rapid ventricular response, respiratory failure with hospital-acquired pneumonia requiring mechanical ventilation, and acute kidney injury requiring hemodialysis (Table 2). Lastly, three postoperative RPN patients developed severe complications. The first patient had grade 3a postoperative AVF required renal angiogram with embolization. The second patient had grade 4a postoperative end-stage renal disease requiring long-term hemodialysis. The third patient had grade 4b postoperative respiratory failure requiring mechanical ventilation and end-stage renal disease requiring long-term renal replacement therapy. Three patients were readmitted. One patient was given intravenous antibiotics to treat an infected intra-abdominal collection after RPN, one underwent angiography and embolization of a renal AVF after RPN, and one underwent angiography and embolization of a renal AVF after OPN (Table 2).

## Discussion

Nephron-sparing surgery by partial nephrectomy (PN) is an appropriate treatment modality for localized renal tumour, especially for T1a renal masses. PN is associated with a substantial reduction in the postoperative risk of developing chronic kidney disease (CKD) linked with radical nephrectomy.^16^ In past decade, RPN has also successfully emerged as a safe and efficacious management option for T1b renal tumors, demonstrating excellent intermediate oncologic and functional outcomes. The European Association of Urology (EAU) guidelines have also advised the use of RPN to preserve renal parenchymal function without compromising it oncologically.^17^ In the past, OPN was the only standard technique. The most common complications after partial nephrectomy, however, are urine leakage, postoperative bleeding, urinary tract infection, arteriovenous malformations, pseudoaneurysms, and renal abscesses.^11^ LPN was later developed as a minimally invasive approach. Ultimately, technological advancement has paved the way for the rise of robotic partial nephrectomy (RPN). As a technically less demanding yet equally effective technique with a shorter learning curve compared to LPN, RPN has been increasingly performed by many hospitals, showing reduced chance of conversion to RN, reduced blood loss, shorter ischemia times and shorter LOS.^18–20^

This single-institute study sought to compare trifecta outcomes and their potential predictive factors in patients undergoing OPN, LPN and RPN at Ramathibodi Hospital. The results did not favor any surgical approach over the others in terms of trifecta achievement. In line with our study, Mehra et al reported no significance difference in trifecta rates among the three modalities.^21^ It is noteworthy that they defined trifecta as a composite of absence of positive margins, perioperative complications and ≤30 min ischemia time, while our definition adopted the minimal decrease in postoperative renal function instead of the ischemia time. A comparative study of open and robotic approaches by Acar et al also found similar rates of achieving trifecta as well as equivalent clinical, surgical and functional results between trifecta-positive and trifecta negative patients.^22^ Likewise, Yerram et al found that achievement of the trifecta outcomes (defined as negative surgical margins, no urologic complications, and ≥90% eGFR preservation at last follow-up) was not different between OPN and RPN, while Zargar suggested that RPN was superior to LPN in this respect.^15^,^23^ A possible explanation is that LPN is inferior to both surgical OPN and RPN because of its more challenging surgical procedure, although such a distinction was not found in our study. Notably, according to Khalifeh et al, trifecta was more easily achieved using the robotic platform.^24^ This is largely due to the improved 3D visualization of the anatomy and better wrist articulation, which facilitates the performance of ergonomically challenging surgeries at higher frequency. Provided that RPN was introduced to our institute in 2015, the robotic-assisted surgical system is still in a relatively nascent stage of operation, and it would be premature to observe substantial trifecta positivity in our RPN cases.

Previously, similarities in postoperative complications such as urine leak and hemorrhage from LPN and RPN were already described by Benway et al.^18^ As in our study, Zargar et al found that the postoperative creatinine concentration and eGFR levels were not different between LPN and RPN performed by various institutes.^23^ Other single institutional studies consistently showed no significant difference in intraoperative outcomes such as blood loss,^25^,^26^ margin status^25^,^26^ and WIT.^27^ Indeed, both Zargar et al and Xia et al reported that the rate of intraoperative complications was similar in OPN and RPN.^28^,^29^ Consistent with our study, Gill et al found that the blood loss was similar for OPN and RPN,^30^ as did Mehra et al for all the three operative platforms.^21^ Nevertheless, Mehra et al and other lines of study suggested the superiority of LPN and RPN over RPN in terms of blood loss, with RPN showing the lowest estimated blood loss.^21^,^31^,^32^ In a similar line of observation, Khalifeh et al^24^ and Porpiglia et al^13^ concluded that the rate of postoperative complications was lower in RPN than in LPN and OPN, although the former did not observe any significant difference in terms of operative time. Crucially, Khalifeh et al found that the operative time was shorter for RPN than LPN,^24^ presenting a contrast with our finding where RPN had the longest operative time. The differing results could be explained by the small sample size in our OPN and LPN groups, the involvement of different surgeons in the operations and the associated learning curves. Particularly, the steep learning curve of RPN and the biased tendency to assign renal masses of greater complexity and higher renal nephrometry scores to the RPN group could plausibly be responsible for its prolonged length of operation. With higher volumes of patients, reduced variability in performance, and increasing surgeon experience, intraoperative blood loss, as well as operative time, is likely to be lower in RPN. As reflected in our results, despite being the most time-consuming procedure, RPN still yielded similar safety and oncologic outcomes as OPN and LPN did. Altogether, our findings suggest that patients undergoing OPN, LPN and RPN could achieve the same perioperative, postoperative and trifecta outcomes.

Trifecta criteria represent a widely recognized assessment of successful partial nephrectomy. To provide a better prediction of both short-term and long-term success following OPN, LPN and RPN, it is thus crucial and interesting to explore which clinical or surgical variables might be associated with trifecta. The existing literature suggests that trifecta achievement is influenced by the type of surgery (RPN), tumor size and complexity, EBL and operative time.^23^,^33^ On the contrary, our study revealed no baseline patient characteristics or perioperative parameters that could predict trifecta outcomes, except for one parameter, LOS. Trifecta and non-trifecta groups differed significantly in terms of LOS and this was further consolidated by the univariate analysis, which pointed to LOS as the only associated parameter of trifecta outcomes. Logically, since one of the risk factors associated with prolonged length of hospitalization is the development of a complicated course, it is not surprising that the patient’s average length of stay would negatively correlate with the achievement of trifecta, whose definition involves the absence of perioperative complications. In line with our study, Acar et al reported LOS as the only parameter that differentiated trifecta-positive from trifecta-negative patients, with the failure to achieve trifecta correlating with longer LOS.^22^ Nevertheless, the median LOS remained similar in our study regardless of the surgical platform, whereas Han et al^34^ and Kim et al^35^ found LOS to be significantly shorter for RPN than OPN. This raises an interesting and relevant point regarding the most cost-effective modality of treatment in the modern era of healthcare where financial resources are a major driver of decision-making in surgery. RPN is known for its high capital and operational costs, while OPN, despite being more affordable in the community setting, is believed to claim longer LOS and more morbidity. Proving that RPN can indeed offer LOS advantages will provide clinical and financial justifications for urologists and patients who are continuously shifting towards robotic treatment for better outcomes. Overall, LOS can be affected by many factors, including patient pain control, patient preference, socioeconomic status, and institutional practice patterns.

Our study encountered three main limitations. First, it was a retrospective, non-randomized study with a relatively small sample size from a single institute. Some of the OPN cases belonged to the pre-robotic era (before 2015), and the greater experience and expertise over time of the surgical teams might render the interpretation of operative outcomes across the treatment modalities more complicated. The small size of the cohort also limited our attempts to match OPN, LPN and RPN cases as well as the statistical power to detect subtle differences between them. Moreover, it would be difficult to randomly assign patients to a specific surgical option without undermining their personal preferences and disregarding their financial concerns. The larger number of patients and proper randomization would therefore improve the accuracy of our comparative study. Second, the multi-surgeon nature of the data collection process may have introduced inherent bias arising from the variability in the learning curves and techniques of LPN and RPN, which could be responsible for a heterogeneous pool of outcomes and predominantly non-significant differences. Third, the postoperative evaluation of eGFR at 1-year follow-up may have proved premature for the determination of actual long-term renal functional changes. An extension of oncologic outcome assessment beyond three years post-operation would improve long-term follow-up studies and offer an extra benefit of screening for signs of renal impairment associated with chronic kidney disease. On the whole, this study provides an insight into contemporary clinical practice and management of renal cell carcinoma in Thai cohorts in the years spanning the transition from the OPN to the minimally invasive LPN and RPN approaches, with the concept of trifecta being used to help redefine the gold standard treatment. Larger prospective and randomized control studies are required to validate our results, and so are studies aiming to analyze the cost-effectiveness of advanced robotic surgeries in low- and middle-income countries with limited economic and technological resources.

## Conclusion

The present study demonstrated that the achievement rate of trifecta was comparable among patients who underwent OPN, LPN, and RPN. Length of hospital stay was shown to be the negatively associated factor for fulfilling the trifecta criteria. In terms of oncological and functional outcomes, the three surgical procedures provided equivalent technical feasibility and safety for the treatment of renal cell carcinoma, suggesting that LPN and RPN may be utilized for the management of small renal masses with complexity in the future.

## Data Sharing Statement

The datasets used and/or analysed during the current study available from the corresponding author on reasonable request.
